# Nutritional Status and Nutritional Support in Patients with Gastrointestinal Diseases

**DOI:** 10.3390/nu17020270

**Published:** 2025-01-13

**Authors:** Beata Jabłońska

**Affiliations:** Department of Digestive Tract Surgery, Medical University of Silesia, Medyków 14 St., 40-752 Katowice, Poland; bjablonska@poczta.onet.pl; Tel./Fax: +48-32-7894251

Gastrointestinal diseases include a wide spectrum of functional and structural disorders of the alimentary system, involving hepatic, bile duct, and pancreatic diseases. Structural gastrointestinal diseases include different inflammatory (inflammatory bowel diseases, as well as acute and chronic pancreatitis) and oncological diseases (contribution 1) [1]. The digestive tract is responsible for ingestion, digestion, and absorption. Gastrointestinal diseases, manifested by a loss of appetite, dysphagia, abdominal pain, and diarrhea, lead to reduced food intake, maldigestion, and malabsorption [1,2]. Additionally, the nutritional status of patients with gastrointestinal disorders is impaired by the adverse effects of their treatment, including anti-inflammatory drugs, chemotherapy, radiotherapy, and surgery. The proper and early detection of malnutrition allows for effective nutritional intervention that improves treatment results [3]. According to current knowledge, malnutrition is reported in 30–50% of hospitalized patients, and in approximately 84% of carcinoma patients. Malnutrition, not cancer, is the cause of death in about 20% of these patients. In patients with gastrointestinal cancers, malnutrition is caused by insufficient food consumption caused by an absence of appetite, dysphagia, disease-related signs (including abdominal pain, nausea, and vomiting), and altered functions of digestion and absorption. In addition, malnutrition is caused by anti-cancer therapies involving surgical treatment as well as radio-chemotherapy. Moreover, hospitalization increases the risk of patient malnutrition (contributions 2 and 3) [3,4,5,6,7,8,9].

In inflammatory bowel diseases (IBDs), involving Crohn’s disease (CD) and ulcerative colitis (UC), protein–energy malnutrition is noted in about 75% of patients with an active disorder. This is related to a reduced food intake due to loss of appetite, maldigestion, and malabsorption, which is secondary to altered digestion and absorption within the small bowel, inadequate food intake, and complications of medical treatment [2,9,10,11]. Regarding medical treatment, glucocorticoids decrease intestinal absorption, leading to deficiencies of phosphorus, calcium, and zinc. Sulfasalazine, as a folic acid antagonist, may lead to folate deficiency and anemia [2]. Micronutrient and vitamin deficiencies are observed in approximately 50% of IBD patients. The most frequent deficiencies involve iron; selenium; zinc; calcium; magnesium; vitamins B6, B12, A, D, and K; and folic acid [9,12]. These deficiencies are more frequently noted in CD compared to UC patients, especially during active disease, and can be related to a greater risk of disease complications [9,13]. Thus, a regular annual clinical and laboratory evaluation of micronutrient deficiencies is recommended by the authors of [2,14]. The British Dietetic Association recommends an evaluation of the levels of folic acid; vitamins B12 and D; iron; zinc; magnesium; and selenium [2,15]. It should be noted that it is not only the above-mentioned conservative treatment used in IBD patients that is associated with malnutrition in this population. Surgical management, such as small and large bowel resections, as well as post-surgery complications, including anastomotic dehiscence, intestinal fistula, and short bowel syndrome, lead to a reduced oral food consumption, altered functions of digestion, and absorption [2]. A deteriorated nutritional status is related to an increased risk of post-surgery complications in IBD patients [2,16,17,18,19,20]. Regarding nutritional interventions in IBD patients, diet modification, vitamin and micronutrient supplementation, and enteral and parenteral nutrition are used. Enteral nutrition (EN) is the first-choice nutritional support in patients with a functional digestive tract, while parenteral nutrition (PN) is administered when the use of EN is not possible [2,21]. It is known that diet regulates intestinal inflammatory processes, whereby it impacts on the bowel microbiome, which, in turn, impacts the permeability of the alimentary tract [22]. As concerns perioperative nutritional support in IBD patients, according to the European Society for Clinical Nutrition and Metabolism (ESPEN), nutritional support, including EN and or PN, should be introduced as soon as possible in malnourished patients undergoing surgery or if an oral diet is not possible within seven days following the surgical procedure. Oral nutritional supplements (ONSs) are recommended when the patient’s energy and/or protein requirements cannot be met by normal food [14]. It has been shown that the routine perioperative administration of ONSs and EN is associated with a lower risk of postoperative infections in IBD patients [14].

Acute pancreatitis (AP) is one of the most common acute gastrointestinal disorders. According to the 2012 revision of the Atlanta Classification, AP can be categorized into mild AP (MAP), moderately severe AP (MSAP), and severe AP (SAP) [23,24]. In AP, especially in MSAP and SAP, inflammatory and septic complications lead to an increased metabolism, energy requirements, and proteolytic metabolism. Additionally, malnutrition is secondary to abdominal pain, appetite absence, and decreased diet consumption. Malnutrition is also associated with an adverse disease course including higher morbidity and mortality, as well as a long duration of hospitalization and increased mortality. Thus, a proper assessment of the nutritional status, as well as early nutritional interventions, are very important in AP patients [23]. Nutritional intervention is required in all SAP patients. It not only improves the nutritional status, but also modulates the deteriorated immune response, as well as preventing gut barrier dysfunction, bacterial translocation, and secondary infectious complications. The oral route of nutritional intervention is the first choice for MAP and MSAP patients. Early EN introduced within 24 to 48 h of admission is optimal for SAP patients with an intolerance to oral food, which is secondary to the severe course of the disease. In SAP patients, EN can be administered via nasogastric tube (NGT) or nasojejunal tube (NJT). In cases of gastroparesis, NJT—not NGT—should be considered because of the higher aspiration risk, pancreatic edema, or large pseudocysts with gastric or duodenal impression. In patients in whom long-term (>30 days) EN is planned, percutaneous gastrostomy or jejunostomy is recommended. PN is indicated only in cases when EN is not possible (contribution 4) [25,26].

Chronic pancreatitis (CP) is a progressive inflammatory pancreatic parenchyma destruction with subsequent fibrosis, which leads to pancreatic exocrine and endocrine insufficiency [26,27,28]. In CP, malnutrition is associated with persistent abdominal pain, alcohol abuse and smoking, decreased food consumption, and pancreatic exocrine and endocrine insufficiency, as well as secondary maldigestion and malabsorption, which manifest as steatorrhea [14]. Micro- and macronutrient deficiencies are reported in CP patients. Therefore, regular yearly laboratory screening is recommended. According to ESPEN, biochemical evaluation should involve measurements of vitamins A, D, E, K, and B12; folate; parathyroid hormone; iron; ferritin; C-reactive protein (CRP); and glycemic control [14]. In CP patients, a restrictive diet is not required. A well-balanced diet is recommended for patients with a normal nutritional status; a high-protein, high-energy diet is recommended for malnourished CP patients. ONSs are recommended for undernourished patients in whom oral feeding is not sufficient to meet calorie and protein requirements. In CP patients with vitamin and macro- and micronutrient deficiencies, which are secondary to malabsorption, adequate vitamin (A, D, E, K, B1, B12, and folate) and mineral (magnesium, iron, selenium, and zinc) supplementation is recommended. EN is indicated for CP patients with malnutrition in whom oral nutritional support is not sufficient. EN using an NJT is recommended in patients with abdominal pain, nausea or vomiting, delayed gastric emptying, and gastric outlet syndrome. Percutaneous endoscopic gastrostomy with jejunal extension (PEG-J), direct percutaneous endoscopic jejunostomy (DPEJ), or surgical jejunostomy are recommended for patients who need long-term EN (>30 days). PN should be considered in patients with ileus secondary to gastric outlet obstruction, the presence of fistulae, or in the case of EN intolerance. In CP patients with PEI, oral pancreatic enzyme supplementation is required [14].

Malnutrition is a frequent problem in gastrointestinal cancer patients [3,7]. It is secondary to a decreased oral diet consumption intake due to dysphagia, odynophagia, appetite absence, ileus, or malabsorption and maldigestion due to exocrine pancreatic insufficiency as a result of pancreatic duct obstruction, jaundice with altered bile secretion and bile flow into the duodenum secondary to common bile duct obstruction, and liver insufficiency. It is also associated with anti-cancer therapy (radiotherapy/chemo-radiotherapy) as a result of its adverse effects including mucositis, dry mouth, taste absence, and dysphagia (contribution 2) [3,7,8]. In addition, major surgery, including esophageal, gastric, intestinal, pancreatic, and liver resections, and postoperative complications, such as pancreatic, biliary, intestinal fistula, and ileus, can lead to the deterioration of nutritional status. A deteriorated nutritional status is related to an increased risk of post-surgery complications. The negative impacts of sarcopenia, decreased muscle mass, and malnutrition on postoperative outcome, including a higher frequency of postoperative morbidity and a longer hospital stay, have been shown. Therefore, preoperative nutritional support, as a part of prehabilitation, is recommended for patients with gastrointestinal cancers undergoing surgery (contribution 5) [3,5,29,30,31,32,33,34,35,36,37,38,39]. According to ESPEN, in severely malnourished patients, surgery should be delayed for 7–14 days to improve their nutritional status [40,41]. An improvement in nutritional status and immune function, as well as a decreased risk of postoperative infectious complications, in gastrointestinal cancer patients receiving preoperative ONSs has been reported. Thus, preoperative ONSs are recommended in patients undergoing gastrointestinal cancer surgery [34]. According to ESPEN, in the perioperative course, in patients in whom meeting energy and nutrient requirements is not possible via oral and enteral feeding alone (<50% of caloric requirement) for >7 days, a combination of enteral and parenteral nutrition is indicated. When EN is contraindicated, PN should be introduced as soon as possible if nutritional support is required [40,41].

In numerous cancers, a systemic inflammatory reaction, appetite absence, and weight loss are reported. The syndrome of reduced appetite, weight loss, metabolic deterioration, and inflammatory status is called cachexia, cancer cachexia, or cancer anorexia–cachexia syndrome (CACS). Deteriorated nutritional status is related to decreased quality of life, increased therapy-related adverse effects, decreased tumor response to anti-cancer therapy, and shorter survival [42]. A proper assessment of nutritional status and adequate nutritional intervention are very important in cancer patients. Nutritional intervention in patients with gastrointestinal cancers involves dietary counseling, ONSs, EN, and PN. Nutritional support is required both in malnourished and well-nourished patients. Initially, nutritional counseling with ONSs is recommended. In patients in whom the oral route is not sufficient to meet energy and protein requirements, EN is recommended (contribution 5) [29,42].

Immunomodulating nutrition, involving glutamine, arginine, omega-3 fatty acids, and nucleotides, via the modulation of immune and inflammatory responses, improves treatment results and patient prognoses, reduces the rate of postoperative infectious complications, and shortens the duration of hospitalization in patients following surgery. IN is indicated for enhanced recovery after surgery (ERAS) in patients following major surgery. Preoperative oral supplementation is also recommended. According to ESPEN, parenteral glutamine and omega-3 acid supplementation may be administered in patients in whom sufficient enteral feeding is not possible [37,40,41].

This Special Issue, entitled “Nutritional Status and Nutritional Support in Patients with Gastrointestinal Diseases”, collects several original and review papers on nutritional status and nutritional intervention in patients with IBD and cancer, as well as other surgical patients.

A single-center retrospective study by Gold et al. [43], including 128 IBD patients, published in this Special Issue demonstrated that the prevalence of food insecurity (FI) was common in IBD patients, which was associated with an increased ultra-processed food intake. Based on this study’s results, the authors recommended the regular screening of IBD patients for FI as a risk factor of malnutrition in IBD patients [43].

In a review paper by Sousa et al. [44], which was published in this Special Issue, the role of selenium (Se) and its deficiency in IBD patients was presented. Se plays an important role in immune system function and its deficiency is related to impaired immune function. The authors presented current knowledge regarding Se intake, absorption, and metabolism, as well as biomarkers of its deficiency and its role in the immune system. Adequate Se consumption is required for the proper functioning of the innate and adaptive immune systems. Thus, Se is an immunonutrient. A Se deficiency is frequently reported in IBD patients, but the current knowledge regarding Se is lacking and numerous issues need to be resolved [44].

In a single-center retrospective study by Yun et al. [45], which was published in this Special Issue, including 116 patients undergoing curative gastrectomy and receiving oxaliplatin and capecitabine (XELOX), the impact of malnutrition on chemoadherence and its subsequent effect on survival was analyzed. The authors demonstrated the significant association between higher Global Leadership Initiative on Malnutrition (GLIM) severity and lower relative dose intensity (RDI). Based on this study’s results, the authors recommended the routine preoperative evaluation of nutritional status using GLIM criteria for the identification of high-risk patients and early nutritional intervention in order to improve chemotherapy adherence and outcomes [45].

In a review paper by Ciesielka et al. [34], which was published in this Special Issue, the association between preoperative sarcopenia and sarcopenic obesity and a risk of postoperative complications in patients undergoing pancreaticoduodenectomy for periampullary malignancies was presented and widely discussed. Sarcopenia, defined as a progressive and generalized decrease in skeletal muscle mass, is often confirmed in patients with various periampullary malignancies. This review demonstrated the significant association between sarcopenia and sarcopenic obesity and a higher frequency of postoperative morbidity, including clinically relevant postoperative pancreatic fistula (POPF) type B and C, as well as delayed gastric emptying (DGE). In addition, a higher rate of infectious complications, post-surgery hemorrhage, and intra-abdominal abscesses was noted in sarcopenic patients [34].

A review paper by Wobith et al. [35], which was published in this Special Issue, regards nutritional prehabilitation in patients undergoing abdominal surgery. Malnutrition plays a crucial role as a modifiable risk factor in patients undergoing major abdominal surgery. Therefore, nutritional support is recommended for malnourished patients and high-risk patients. The association between preoperative nutritional support and a reduced risk of postoperative complications has been reported. Currently, nutrition is a part of multimodal prehabilitation. In this review, an evaluation of nutritional status and indications for prehabilitation, the duration of nutritional prehabilitation, and various nutritional interventions, including dietary counseling ONSs and immunomodulating nutrition, are presented [35].

In conclusion, an evaluation of nutritional status and proper nutritional support are very important in numerous inflammatory and oncological gastrointestinal diseases. First, the deterioration of nutritional status is secondary to the dysfunction of the alimentary tract due to a disease-related loss of appetite, dysphagia, odynophagia, ileus, malabsorption, and maldigestion. In addition, conservative anti-inflammatory and oncological treatment, as well as major surgery, deteriorate nutritional status in patients with gastrointestinal diseases. Therefore, nutritional support during basic disease treatment, to prevent malnutrition, is required as soon as possible. It should be noted that nutritional support is a very important and integral part of the management of patients with gastrointestinal diseases.
